# Exercise Physiology, Cognitive Function, and Physiologic Alterations in Extreme Conditions

**DOI:** 10.1155/2015/359325

**Published:** 2015-03-25

**Authors:** Ellen Glickman, Edward J. Ryan, David Bellar

**Affiliations:** ^1^School of Health Science, Exercise Physiology, Kent State University, Kent, OH 44242, USA; ^2^Exercise Science, Chatham University, Pittsburgh, PA 15232, USA; ^3^School of Kinesiology, University of Louisiana-Lafayette, Lafayette, LA 70506, USA

*Exercise physiology* is a heterogeneous field of study that includes a broad array of disciplines evaluating the impact of physical stressors on the physiology of man. While man is exposed to a variety of environmental conditions, it is imperative that exercise physiologists elucidate how the added stress of a terrestrial extreme impacts man's thermal, metabolic, and cognitive abilities. Research regarding environmental extremes and exercise will enhance our understanding of how to safely compete athletically, navigate in unfamiliar locations, and aid military personnel during exposure to a variety of natural settings.

The physiologic, metabolic, and cognitive responses in general are complex and differ between sexes, across the lifespan, and are impacted by heredity. When physical stress is coupled with varied ambient conditions (environmental stressors, i.e., heat, cold, altitude, hypoxia, and lower body negative pressure), there may be dramatic shifts in physiological and cognitive responses. Furthermore, this coupling of stressors may present a limitation in man's abilities to maintain homeostatic control across multiple organ systems. Such limitations may compromise safety and performance; thus further work in these areas is warranted.

Through controlled experimentation, taking into account as many confounding factors as possible (i.e., gender, menses, age, training status, circadian rhythm, and diet), we evaluate combined physiologic and cognitive responses to environmental and exercise stresses. Recently, there has been growing concern for works elucidating how heat/cold stressors influence blood flow, cognitive function, and thermoregulation at rest and during exercise. While military personnel are often deployed to areas of low barometric pressure, research has also been conducted to better understand the effects of hypoxia on cognitive function.

We invited investigators to contribute original research articles as well as review articles that will stimulate the continuing efforts to understand exercise physiology in ambient and extreme environmental conditions. Further research in these areas will allow for better precautionary and treatment guidelines in occupational and athletic settings.

*Ellen Glickman* *Edward J. Ryan* *David Bellar*

